# Comparing Approaches to Research in Global and International Health: An Exploratory Study

**DOI:** 10.5334/aogh.2799

**Published:** 2020-04-29

**Authors:** Kristy C. Y. Yiu, Eva Merethe Solum, Deborah D. DiLiberto, Steffen Torp

**Affiliations:** 1 Global Health Office, Faculty of Health Sciences, McMaster University, CA; 2 Department of Health, Social and Welfare Studies, University of South-Eastern Norway, NO

## Abstract

**Background:**

Global health is a term often used interchangeably with international health due to overlapping similarities and unclear distinctions. While some international health supporters argue that global health as a field is unnecessary as it is simply a duplicate of international health, global health supporters argue that global health is unique; for instance, it actively includes elements of empowerment and promotes cross-border collaboration.

**Objective:**

To investigate differences and similarities in research representing the fields of global and international health.

**Methods:**

We analyzed all the articles published in 2017 in two comparable academic journals representing the fields of global health (*Annals of Global Health*, AGH) and international health (*International Health Journal*, IHJ). Abstracted data included: research design and methods, income status of country of study, empowerment recommendations for practice, participation and research collaboration.

**Findings:**

Most studies in both AGH and IHJ used quantitative research methods but were significantly more common in IHJ (70%) compared to AGH (48%), whereas mores studies in AGH (17%) than IHJ (9%) used mixed methods. The majority of studies in both journals focused on low- or lower-middle income countries whereas more AGH studies (16%) focused on high-income countries compared to the IHJ studies (4%). It was more common in the AGH studies to make empowerment recommendations (90%) and to include stakeholders/users in the study (40%) compared to the IHJ studies (75% empowerment recommendations and 18% stakeholder/user participation). No difference was observed regarding cross-border research collaboration.

**Conclusions:**

This study does not show great differences between global health and international health research; however, there are still some differences indicating that global health emphasises different aspects of research compared to international health. More research is necessary to understand whether and how the distinctions between the definitions of global and international health are applied in real life, in research and beyond.

## Introduction

‘Global health is fashionable’ [1]. This quote suggests that the emergence of ‘global health’ as a popular concept could be easily replaced and overshadowed, similar to the process observed with its predecessor, ‘international health’ [2]. Since its emergence, some have observed global health as the preferred authoritative term for universities, government agencies, and private philanthropies [3]. This may stem from the fact that global health is welcomed by parties from extremes of the political spectrum [4]. The perception that global health could replace international health implies that the two share exchangeable commonalities. This may be due to the very same similarities, as well as the lack of a commonly agreed upon definition of global health, that has led to the ongoing debate on differences between global health and international health in the scientific community. Specifically, the discussion revolves around whether there is a need for global health when international health has already adequately covered the study and practice of cross-borders health issues [5].

The confusion between the terms is unsurprising. Global health, a concept emerging from the late 1980s [6], is seen to be derived from international health, and both are derived from the greater field of public health. The highly cited article by Koplan et al [1]. demonstrates that the three entities – public, international, and global health – share a number of characteristics including: prioritizing a population-based and preventive focus; concentrating on poorer, vulnerable, and underserved populations; adopting multidisciplinary and interdisciplinary approaches; emphasizing health as a public good and the importance of systems and structures; and encouraging the participation of multiple stakeholders.

Despite these commonalities, global health possesses unique features that are not demonstrated in international health, including issues of globalization, focus on scope of problem that is not dictated by geographical boundaries, aim of equity in health for all, empowerment of the local community, and advocacy. In fact, global health advocates maintain its representation as a unique sphere in health care. As seen by the increasing interdependence of people and countries around the world as a result of ‘cross-border trade in goods and services, technology, and flows of investment, people, and information,’ it could be seen that globalization is on the rise [7]. In light of this globalization trend, global health places particular focus on these issues.

In close association to this characteristic, global health also refers to the scope of the problem as opposed to the location of the problem, as international health does [1,4,8]. In other words, global health recognizes that health problems are not bounded by national borders. Global health is not dependent on the socioeconomic status of a nation and does not necessarily have to take place in low- and middle-income countries, as opposed to international health, where it gives exclusive focus to low and middle income countries [9]. In this way, domestic health problems of a high income country (HIC) could also fall under the scope of global health. As an indicator of equitable research, geographical reach could also be applied in the comparison of the location of the authors’ affiliations to the study location. If either of the main contributors of the study (i.e., first or last author) originate from a country whose income status is equivalent to or lower than that of the country of study, then the study could be seen as equitable research in this regard. However, if both of the main contributors of the study originate from a country whose income status is higher than that of the country of study, the study might not be seen as equitable in this regard.

The aim of equity in health for all is another key feature of global health [1,10]. In regards to global health, it does not only pertain to health outcomes, as demonstrated in international and public health, but also encompasses the aim of achieving equity in the processes of realizing the outcomes [1,10]. In other words, in accordance to global health proponents like Brown and Closser [4], or organizations like the Canadian Coalition for Global Health Research [11], global health encourages the development of an equitable and non-paternalistic partnership between international donors and stakeholders and local communities.

As well, global health also advocates for the empowerment of the local community. Empowerment has been defined by the World Health Organization (WHO) as ‘a process through which people gain greater control over decisions and actions affecting their health [12].’ Thus, empowerment of the local community could be demonstrated through active inclusion of community members in the research process [13]. This focus on the local community is in line with the argument that global health encourages the increase of influence of non-governmental organizations as well as the decrease in the influence of international organizations [4]. In close relation to empowerment, advocacy is also mentioned in relation to global health, although it is seen to still be in its infancy [14]. Furthermore, global health advocacy is often described in the context of creating political change to advance global health equity [15]. However, this does not mean that global health research has no part to play in global health advocacy. On the contrary, Basilico et al. [15] greatly emphasizes the need for global health research to provide evidence-based support for policies. One way to inform policymakers could be through recommendations proposed by researchers in their papers. Moreover, recommendations targeted at the local community could be an indication of the researchers’ desire to empower community members through actionable suggestions informed by research.

Despite the numerous distinctions offered by global health supporters, there are advocates for international health that claim that these differences are implicitly encompassed in the definitions or mission statements of their fields. If there are any distinctions between global and international health in research, it would be expected that they are reflected in research deliverables, such as published journal articles. Still, to our knowledge, no study has investigated whether there are any practical and/or scientific differences between the two fields in the realm of research. Therefore, the aims of this exploratory study were to investigate differences and similarities in research representing the two fields and to examine whether key aspects of global and international health research align with their respective literature definitions.

Beyond gaining a better understanding of the two fields in research, our findings will allow us to examine what is currently researched in global health and international health respectively. In turn, this will inform us of the topics of research that are currently valued.

## Methods

The conceptual model of this study was mainly built upon Koplan et al.’s [1] framework in comparing global, international, and public health. We conducted an exploratory study, which is defined by Hallingberg et al [16]. to be ‘studies intended to generate evidence needed to decide whether and how to proceed with a full-scale effectiveness study.’ This exploratory study examined approaches to research in global health and international health reported in scientific peer-reviewed articles published over the course of one year (2017) in two comparable academic journals representing the fields of global health and international health.

### Journal selection

In addition to browsing a list of popular journals from the two fields, as well as using our knowledge of the subject areas, we searched the internet for any global health and international health academic journals. Potential journals were compared using the following criteria:

- peer reviewed,
- full year of publication in 2017,
- written in English,
- frequency of publication,
- affiliation(s) with academic or public institutions,
- publisher and its location,
- impact factor, and
- open access.

The criterion of having a full year of publication in 2017 ensured that there would be a sufficient number of articles in each journal for evaluation. At the same time, only one year of publication was chosen as it kept the volume of papers for review manageable. The frequency of publication was considered to ensure that both journals will have a similar number of articles for evaluation. Given that English is the common language among the study authors, the selected journals must be written in English for our evaluation. Journals are often affiliated with academic or public institutions in the same fields or possess a similar interest. Thus, examining the journals’ affiliation(s) with academic or public institutions could provide us with further understanding of the journals’ establishment in the global health or international health fields. In recognition that the majority of the research literature is dominated by researchers originating from the Global North, we considered the location of the journals’ publishers [17]. The impact of an academic journal is often measured by its impact factor and this measurement was also used as a criterion when selecting two journals of similar caliber in global health and international health for comparison. Lastly, given the nature of both global health and international health, and that many of these research collaborations would include researchers from the Global South, it was crucial that there are no financial barriers to accessing research papers. Therefore, accessibility was also another major consideration and eligible journals should have open access. Our search results included eleven eligible global health journals and seven international health journals. After comparing all 18 journals according to these aspects, we decided to use the *Annals of Global Health* (AGH) [18] and *International Health Journal* (IHJ) [19] as the two journals for comparison in our study because these two were the most similar with regards to the eight aspects mentioned above. From a global health and empowerment perspective, it is interesting to see whether the selected journals differed with regards to what kind of countries were represented in the editorial teams and also with regards to the availability of financial support for publication fees. We investigated these two issues and found that the two editorial teams of AGH and IHJ were rather similarly composed as respectively 85% and 84% of the editors were based in HIC. Also, both journals offered waivers and discounts for publication fees, although it should be noted that IHJ only offered this financial support to corresponding authors based in low-income countries (LIC) and middle-income countries (MIC) while no such restriction applied in AGH. The two journals are further described in Table 1. The journals included for comparison are described in Appendix A.

**Table 1:** Description of *Annals of Global Health* and *International Health Journal*.

Journal	Affiliation	Publisher (Location)	Impact Factor (1-year)	Open Access	Frequency of Publication	Composition of Editorial Team	Financial Support for Publication Fees

*Annals of Global Health*	Not affiliated with an organization	Levy Library Press (UK)	1-year: 1.833	Hybrid	6 times per year	HIC: 85% (41/48) UMIC: 8% (4/48) LMIC: 4% (2/48) LIC: 2% (1/48)	Authors without funds to pay may request for discount or full waiver
*International Health Journal*	Royal Society of Tropical Medicine and Hygiene	Oxford Academic/Oxford University Press (UK)	1-year: 1.784	Hybrid	6 times per year	HIC: 84% (32/38) UMIC: 8% (3/38) LMIC: 3% (1/38) LIC: 5% (2/38)	Waivers apply for corresponding authors from LIC and MIC and those in genuine hardship

Abbreviations: HIC: high-income country; LIC: low-income country; LMIC: lower-middle-income country; MIC: middle-income country; UMIC: upper-middle-income country

### Data extraction

After the elimination of conference abstracts, editorial articles, and creative literary pieces (e.g., poems), there were 60 articles available for extraction in the AGH and 44 in the IHJ. All of the articles were published in 2017. The following data were extracted from all 104 eligible articles.

#### Types of study

The following types of papers were included in the study: interventional study, observational study (including case study), economic analysis (e.g., cost-benefit analysis), literature review, discussion article, modeling, and tool/program development/evaluation/description. Interventional study/trial is defined as a prospective research study that assigns participants to one or more interventions, such as drugs, biological products, surgical procedures, radiologic procedures, devices, behavioural treatments, preventive care, to evaluate their effects on health outcomes [20]. Observational study is one in which participants are observed and measured for certain outcomes, but as opposed to interventional studies, researchers do not intentionally affect the outcome [21]. Economic analysis measures the economic outcomes of an initiative and with regards to healthcare research, these analyses are primarily designed to maximize the value of the health service or intervention [22]. Literature review is an evaluative report consisting of information found in the literature and aims to describe, summarize, evaluate, and clarify the literature in a particular area of study [23]. Discussion articles include commentaries and opinion pieces, and may be a critical challenge to an article, an extension of the position stated in an article, or an application of a theoretical or methodological perspective [24]. Due to the nature of international and global health, the type of modeling paper in question refers to predictive models, which are used to describe an abstract or hypothetical behaviour or phenomenon or to project health outcomes [25]. Program evaluations are mainly narrative in nature and aim to examine the processes and outcomes associated with the program in question [26]. It could either be formative research where the goal is to improve the current program or summative research where it evaluates its effectiveness [26].

#### Research methods

We extracted information on the type of research method employed in each article, which could be quantitative, qualitative, or mixed methods.

#### Income status of country where study has taken place

To determine whether the notion that international health gives exclusive focus to low- and middle-income countries holds true in research, we extracted the information on the country in which the study has taken place and categorized them as high- (HIC), upper-middle- (UMIC), lower-middle- (LMIC), and low-income country according to the list of countries’ incomes published by the World Bank [27].

#### Topics of research

Using an article released by the WHO titled ‘Ten Threats to Global Health in 2019’ as a basis [28], we categorized the topics of research into: HIV/AIDS, high-threat pathogens (e.g., Ebola), non-communicable diseases, systems-based, children’s health, women’s health, refugees’ health, education, socio-economic-related issues, and others.

#### Formulation of recommendations as an indicator of empowerment

This is another characteristic of global health that is not explicitly present in the definition of international health [5]. To determine whether an element of empowerment is present in the research, we looked for the presence of recommendations in the discussion and conclusion sections of the article. If recommendations were present, we further examined whether they were directed towards a specific organization or set of stakeholders, or if they were general recommendations that did not pertain to any particular audience. If the former applies, then it may suggest that the authors were specifically aiming to use their research to empower those specific stakeholders.

#### Participation of stakeholders or population in research study as an indicator of empowerment

For the purposes of our study, subjects’ involvement in data collection did not count as collaboration as they were not actively contributing to the methods and approach of the study, but rather simply providing information required for analysis. In our analysis, we have made the distinction between participation through collaboration or involvement in data collection. As described above, there are two major key characteristics prominent in global health – equity in partnership between researchers, stakeholders and the community and the empowerment of partner and community. To identify the presence/intent of an equitable partnership between the researchers and the community, we first determined whether the relevant stakeholders were included in the planning and execution of the study beyond the purposes of data collection. The introduction and methods sections were used to assess community participation (e.g., citizen panels or group discussions that assist in determining the research direction).

#### Extent of research collaboration between institutions/countries

There are multiple aspects to achieving an equitable partnership and one of the means to do so is through merit-based authorship [29]. In other words, the order of author listing is determined by the individual’s relative contribution to the research. To identify the presence of an equitable partnership between researchers of the different countries, we examined the author list of each study, particularly the first and last authors as they are usually the researcher who contributed the most and the senior member of the research team respectively [30]. The representation of authorship has been deemed to be an important means of giving individual researchers credit where it is due [30]. Thus, a study aimed towards an equitable partnership should include both external and local researchers, and their contributions to manuscript writing should be reflected appropriately on the author list.

#### Income status of affiliated countries of first and last authors and countries of study

In a similar vein as the last trait, this characteristic takes it a step further by considering the socioeconomic status of the countries of the main authors and of the country under study. With the danger of HIC researchers conducting extractive research at the expense of the local low- and middle-income country communities, equitable research is an essential component of global health research [31]. Extractive research is seen as HIC researchers using communities in low- and middle-income countries for the purposes of data collection then claiming the recognition and benefits of the published research. This is a common threat as HIC scientists usually provide most of the funding and thus often would dictate the research agenda [32]. In this sense, it would be expected that in extractive research majority of the authors would originate from HIC as that would allow them to claim the recognition of the publication; and on the other hand, less recognition, as demonstrated by the smaller proportion of authorship, would be accredited to researchers from low- and middle-income countries. In contrast, there should be a similar proportion of authors from low- and middle-income countries and HIC in publications representing equitable research. Furthermore, Chu et al [31]. described that the transfer of research skills from HIC to low- and middle-income country partners is a key goal of global health research collaboration, thus it is pertinent that collaborators from low- and middle-income countries are well represented in journal publications. Given this rationale, if the first or last author is affiliated with an institution in a country with the same income status as that of the country of study, then the study is considered to be equitable.

### Data analysis

First, we read the abstracts of all the studies and excluded the types of studies we did not want to include in the analysis (e.g., creative literary pieces). Consensus was reached among the authors before proceeding further. Second, we extracted the relevant information from the 104 articles and organized them into predefined categories in a template-organizing style [33]. We also accommodated for some new categories that emerged from what we read. Although we present the results of this study in quantitative terms, such a review of articles is partly based on qualitative analysis, which includes a hermeneutic approach based on the researchers’ previous understanding of the topic. Using *t-*tests [T = p/rota p(1–p)], we have also calculated the significance level of the differences of various outcome variables between the two journals using the free statistical package Zigne [34]. The significance level was set at 5%.

## Results

The results of this study are detailed in Table 2.

**Table 2:** Description of articles published in 2017 in *Annals of Global Health* (n = 60) and *International Health Journal* (n = 44).

Variable	Annals of Global Health, n (%)	International Health Journal, n (%)

*Type of study*		
Interventional study	1 (2)	1 (2)
Observational study	34 (57)	23 (52)
Financial analysis (e.g., cost analysis)	2 (3)	3 (7)
Literature review	7 (12)	3 (7)
Discussion article	2 (3)	2 (5)
Modeling	0 (0)	2 (5)
Tool/program development/description	14 (23)	10 (23)
*Research methods*		
Quantitative methods	29 (48)**	31 (70)**
Qualitative methods	16 (27)	4 (9)
Mixed methods	10 (17)*	4 (9)*
N/A	5 (8)	5 (11)
*Income level of country/countries of study^1^*		
Low-income country	11 (16)	9 (20)
Lower-middle-income country^2^	17 (25)	13 (28)
Upper-middle-income country	14 (21)	10 (22)
High-income country	11 (16)*	2 (4)*
N/A (e.g., review, global/regional study)	15 (22)	12 (26)
*Topic of study*		
HIV/AIDS	2 (3)*	6 (14)*
High-threat pathogens	3 (5)*	9 (19)*
Non-communicable diseases	6 (10)	8 (17)
Systems-based	5 (8)	8 (17)
Children’s health	18 (30)**	5 (11)**
Women’s health	3 (5)	2 (4)
Refugees’ health	0 (0)*	3 (6)*
Education	13 (22)**	0 (0)**
Socio-economic related	4 (7)*	0 (0)*
Others (e.g., ethics, modeling, travel)	6 (10)	3 (6)
*Presence of empowerment recommendations*		
Yes – definitive	12 (20)	10 (23)
Yes – through broad recommendations	42 (70)*	23 (52)*
No	6 (10)*	11 (25)*
*Participation of stakeholders/users*		
Yes – collaboration^3^	8 (13)	3 (7)
Yes – data collection	16 (27)*	5 (11)*
No	36 (60)**	36 (82)**
*Extent of research collaboration*		
Yes – collaboration with researchers from other countries	32 (53)	24 (55)
No – no collaboration with researchers from other countries (researching own country + others/global)	20 (33)	17 (39)
No – no collaboration with researchers from other countries (researching only another country)	8 (13)	3 (7)
*Income status of affiliated countries of first and last authors and countries of study*		
Not applicable – study is not country-specific	12 (20)	9 (20)
Equitable – either first or last author is based in a country at the same income status as the country of study	26 (43)*	28 (64)*
Unequitable – neither first nor last author is based in a country at the same income status as the country of study	22 (37)**	7 (16)**

* p < 0.05, ** p < 0.01 (difference between journals). ^1^ The numbers do not match the total number of articles included as some studies involve multiple countries. The percentages for this section are calculated using the sum of all countries that participated in the study (68 for Annals of Global Health and 44 for International Health Journal) instead of the sum of all included studies. ^2^ Studies that focus on low- and middle-income countries in general, without specification of countries, are counted as a single entry towards the total sum. ^3^ In collaboration with stakeholder in planning or implementation or primary researcher is stakeholder.

### Types of study

There were small differences between the two journals in the kinds of studies that were published during 2017. Observational studies were most common in both journals with AGH (57%) publishing more articles of this type when compared to IHJ (52%). Twenty-three percent of the articles were studies on development or evaluation of a program or tool.

### Research methods

With regards to research methods, both journals had published the most studies using quantitative methods. Still, there is a statistically significant difference between the two journals as 48% of the published studies in AGH had used quantitative methods whereas as many as 70% of the published studies in IHJ had used such research methods. The percentage of studies that used qualitative methods were for AGH 27% and IHJ 9% and for mixed methods 17% and 9% respectively (p < 0.05).

### Income level of country/countries of study

When examining the country/countries in which the studies had been conducted, 41% of the articles published in AGH were conducted in LIC (16%) or LMIC (25%) while 48% of the articles published in IHJ had been conducted in such countries (20% and 28% respectively). When it comes to studies performed in HIC there was a significant difference between the two journals showing that 16% of the AGH articles reported on studies in HIC whereas it was only 4% in IHJ.

### Topics of research

Significantly more AGH articles focused on children’s health and on global health education compared to the IHJ articles as more than 50% of the articles published in AGH focused on these two topics (30% and 22% respectively). On the other hand, significantly more of the articles published in IHJ focused on HIV/AIDS (14%) and high-threat pathogens (19%) when compared to articles in AGH.

### Formulation of empowerment recommendations

Approximately one-fifth of the studies in both journals (20% and 23%) focused explicitly on empowerment. Still, significantly more articles in AGH (70%) provided broad recommendations about empowerment compared to the IHJ articles (52%). Overall, only 10% of the AGH articles did not focus on empowerment in one way or another compared to 25% of IHJ articles (p < 0.05).

### Participation of stakeholders/users

Overall, there were significantly more studies published in AGH (40%) compared to the IHJ articles (18%) that had included stakeholders or users in the study. It was a similar pattern for both collaboration (13% and 7%, respectively) and data collection (27% and 11%, respectively), but the difference was only statistically significant for data collection.

### Extent of research collaboration

In both AGH and IHJ, slightly more than half of the articles (53% and 55%, respectively) reported that there was a research collaboration between researchers from two or more countries.

### Income status of affiliated countries of first and last authors and countries of study

Of the articles that focused on a particular or a particular set of countries (i.e., not a global study), IHJ consisted of significantly more articles (64% vs. 43% in AGH) where the income status of the countries with which the first and last authors are affiliated is the same as that of the country/countries under study.

## Discussion

In an attempt to contribute to the larger discourse on how to define and understand what global health is or should be, the aim of this exploratory study was to investigate whether research within the disciplines or fields of global health and international health differ in accordance with how they are defined [1]. Our study results indicate that there are some differences; even in only comparing the overall characteristics of the two journals, we can gain insight in their tendency to pursue a more global health versus international health direction. When compared to the IHJ studies, the AGH studies used less quantitative research methods and more mixed methods, were more often performed in HIC, and were more focused on users including elements of empowerment and including stakeholders and in their study process.

The composition of the two editorial teams were quite similar, with majority of the editors based in HIC. This is a somewhat unexpected finding for AGH as it would be presumed that a journal based in a discipline that advocates for research equity would consist of more editors from low- and middle-income countries. This finding demonstrates that the operations of journals based in a specific discipline may not necessarily embody the key characteristics of said discipline.

With regards to financial support for publication fee, both AGH and IHJ offer waivers and discounts. However, it should be noted that IHJ imposed a restriction whereby this offer is only available to corresponding authors who are based in low- and middle-income countries. This aligns with our understanding of international health which places specific focus on low- and middle-income countries. Meanwhile, AGH offers financial support to any authors without sufficient funds. This also aligns with our understanding of global health, which does use geographical boundaries as a criterion in its definition.

The articles representing the two fields did not differ regarding type of studies. This finding is in line with the available definitions and comparison of the two fields in the literature, as none of them discerns between the two fields using the type of study produced from research as a criterion. From our results, it could be seen that there is a difference between the types of research methods most commonly used in the two disciplines. While this does not mean that research methods is a crucial factor that distinguishes between the two, nor should the definitions be updated to reflect this, this is still a noteworthy finding as it hints at the epistemology and the types of collaboration with other disciplines that researchers in international health or global health are most likely to form.

Given the available definitions of international health, we expect that majority of the studies from IHJ would focus on countries with a lower socioeconomic status. This assumption holds true as nearly half of the studies take place low- and middle-income countries, but this is also the case for the global health studies. Based on the definitions of global and international health [8], we had expected a starker difference regarding the focus on low- and middle-income countries. Nevertheless, we found that the AGH focused significantly more often on health issues in HIC, such as global health education for HIC trainees. IJH studies focused more on infectious diseases, which are more prominent in low- and middle-income countries and favoured by external funders. International funding plays a huge role in dictating the direction of research and one of the most notable donors since the early 2000s has been the Global Fund to Fight AIDS, Tuberculosis, and Malaria [35,36]. This was in line with the significantly higher proportion of IHJ articles that focused on HIV/AIDS and other high-threat pathogens. Overall, this was in accordance with our expectation as it has been held that global health should have a focus on health-related consequences of globalization that pertain to all countries rather than on health issues solely in countries with low socioeconomic status [9].

We included two different measures for empowerment: study recommendations and participation of stakeholders and/or users in the study. The AGH studies included broad recommendations regarding empowerment more often than the IJH studies, and the AGH studies also included stakeholders/users more often in their work. Although more studies in AGH argued for empowerment through broad recommendations when compared to those published in IHJ, this general feature of empowerment was still highly visible in the articles from both the journals (90% of the AGH studies and 75% of the IHJ studies). Empowerment is most commonly portrayed through active inclusion of stakeholders and community members. However, as a unique element to research, researchers can also express their desire to empower the population through their recommendations in research papers. However, it should be noted that this element of empowerment is not explicit in most cases (70%), but rather vaguely referred to in its broad and generalized recommendations that are not tailored to a specific audience in mind. This finding is supported by Brown et al. [37], who noted that most recommendations in research are general and less than helpful, and further concluded that ‘the potential value of these recommendations is lost’ (page 804). In most cases, recommendations are used to address limitations in current research and pose alternatives to overcome them in future work, either as continuation or long-term development of the current study [38,39].

Research equity with regards to geographical composition of the study authors was also examined. This was measured through comparing the income status of affiliated countries of first and last authors and countries of study. Compared with AGH, IHJ included a higher proportion of articles that had first or last authors whose country/countries of affiliation is that same of that of the country under study. This finding does not align with our understanding of global health, as its advocacy for equity should also extend to creating opportunities for researchers from low- and middle-income countries as well as those from HIC. Echoing the findings from Kerasidou’s study [40], our results also show the need for policies that would shift more power from researchers based in HIC to those based in low- and middle-income countries, thus enabling them to establish collaborations as equal partners. Such a shift in power dynamic should be reflected in authorship credit; however, this was not seen in our findings. That being said, the general trend of inequity in authorship credit may not be exclusive to global health, but rather is a part of a greater phenomenon where the proportion of first authors from non-low- and middle-income countries outweighs that of first authors from low- and middle-income countries [41]. In a study conducted by Kelaher et al. [41] which examined trends in first authorship of randomized controlled trials (RCT) in HIV/AIDS, malaria and tuberculosis conducted in low- and middle-income countries they found that low- and middle-income country first authorship was more likely when the study was financed by low- and middle-income country funding as opposed to non-US HIC funding. Given the findings of their study and ours, it may be worthwhile to conduct a similar study on global health studies, not restricted to RCTs, to see whether these trends of low- and middle-income country first authorship in association with funding sources are currently applicable.

Although the definition of global health portrayed in the literature [1,4] is an active advocate for empowerment, this was in fact rarely demonstrated in any of the studies that we examined. Only 13% of the AGH studies and 7% of the IHJ studies actively included their stakeholders and users in their study beyond the means of data collection. The call for empowerment through community engagement is not novel and not exclusive for global health, but instead was established prior to the emerging era of global health, most notably in the Alma-Ata Declaration of 1978 [42]. Despite the good intentions of the Declaration, Lawn et al. [43] showed in their review of global policies that this has not been translated into action, which is echoed in our findings. As a means of encouraging more researchers to place an emphasis on empowerment in their research, support and findings on the impact of empowerment on research and on the community would be useful. In addition, given how certain empowerment-based research areas, such as participatory action research, have become ‘fashionable’, the need for further research on empowerment is pertinent.

### Limitations

The most important limitation of this exploratory study is that the studies published over the course of one year in the two selected journals were not necessarily representative for all studies defined as global health or international health studies. Still, as this study was partly based on a qualitative and quantitative analysis, it provides some indications on differences and similarities between the two fields. Thus, it contributes to the on-going discussion on whether there are any differences between the two fields and whether the newer discipline of global health adds something more, or rather a different type of research, into the field of public health from a global perspective.

We used the authorship list, namely the affiliation and location of each author, as an indication of collaboration, strength of partnership, and equity in research. However, we recognize that the author list is not wholly representative of everyone involved in the studies, as only the main researchers in planning and writing the manuscript are given credit. Thus, it may not accurately represent the full extent of cross-border collaboration. Furthermore, most scientific journals publish in English, thus non-English speaking researchers may partake in a role that is less involved in manuscript writing. In addition, as our information on each study is solely reliant on the text provided in each article, it may not capture aspects of the study that were not explicitly written as each journal imposed a strict word limit for publication. This may have prompted the authors to omit less important sections, such as participation of stakeholders or encouragement of empowerment, as they may not be recognized to be crucial in the standards of conducting studies and writing papers. Still, these restrictions to our analysis are similar to both of the selected journals.

Furthermore, given the overlap of global health and international health studies in the two journals, this suggests a possibility of the factor of randomness at play. For example, a study may satisfy the criteria set out by both the global health and international health journals, but the journal’s decision to accept it as a publication may consolidate its status as either global health or international health.

## Conclusion

Although this study does not show great differences between global health and international health research on parameters relevant for the definition of the two different fields, there are still some differences indicating that global health emphasises different aspects of research compared to international health. This gives support to those who argue that international health does not adequately address important aspects that are the focus of global health, in particular with respect to aspects of empowerment and equity in partnership. This also supports the argument that the focus of globalization in global health is framed as something more than, or different from, international health’s focus on health issues in low- and middle-income countries. In addition, it seems that the global health research is more diverse when it comes to research methods.

We do not want to take a stance on whether it is useful to make a distinction between the two fields or not, but we observe that in medicine, the evolution of our current-day specializations in healthcare has manifested in a pragmatic manner [44]. Disciplines are flexible, malleable in adapting to the demands of the changing landscape in real life, as well as what their users need. It is plausible that the demands of an ever-changing landscape will influence the development of the scientific fields of global and international health as well. Whether these changes will lead to distinct differences or more similarities between global and international health is still unclear. More research, such as more detailed content analyses on papers describing the distinctions between global and international health, is necessary to understand whether and how the distinctions between the definitions of global and international health are applied in real life, research, and beyond.

## Additional File

The additional file for this article can be found as follows:

10.5334/aogh.2799.s1 Appendix A. Characteristics of global health and international health journals included in journal selection process.
