# Supplementary material for: Comparing Approaches to Research in Global and International Health: An Exploratory Study
Source: Ann Glob Health. 2020 Apr 29;86(1):47. doi: 10.5334/aogh.2799 (PMC7193757; doi:10.5334/aogh.2799)
Supplement: Appendix A. — Characteristics of global health and international health journals included in journal selection process. [file agh-86-1-2799-s1.pdf]

Appendix A: Characteristics of global health and international health journals included in journal selection process

Global Health

Journal name	Affiliation	Publisher	Impact factor	5-year impact factor	Open access	Frequency of publication	Financial support for publication fees
Journal of Global Health (JoGH)	Edinburgh University	Edinburgh University Global Health Society (UK)	2.804 (2016)/ 4.195 (2017)	N/A	Yes ($1200 fee for research articles and $600 for non-solicited viewpoints)	Twice a year	Fees waived for authors from developing countries without sufficient funds
BMJ Global Health	N/A	BMJ Publishing Group (UK)	N/A	N/A	Yes	Quarterly (4 issues + 4 supplementary)	Unfunded authors from low-income and lower-middle-income countries may claim waiver or discount
The Lancet	N/A	Elsevier	17.686	18.248	Yes	Monthly	Authors whose main funder

Global Health		(Netherlands)	(2016)	(2016)			is located either in group A or B countries of the Health Inter Network Access to Research Initiative (HINARI) or in a country with a low UNDP human development index are exempt
Global Health Promotion	International Union for Health Promotion and Education	SAGE Journals (UK)	1.442 (2016)	N/A	OnlineFirst (forthcoming articles are published online before they are scheduled to appear in print)	Quarterly	Not offered
Global Health	N/A	BMC part of	N/A	N/A	Yes	No schedule	Authors based in countries

Research and Policy		Springer Nature					classified as LIC or LMIC by World Bank may request waiver or discount
Annals of Global Health (previously The Mount Sinai Journal of Medicine)	N/A	Levy Library Press (UK)	1.833	N/A	Hybrid	6 times a year	Authors without funds to pay may request for discount or full waiver
International Journal of Global Health	N/A	SciTechnol (UK)	N/A	N/A	Yes	Unknown; they have yet to publish their first issue	No publication fees. Open access is optional but did not mention whether fees could be subsidized
Journal of Epidemiology and Global Health	Saudi Ministry of Health	Elsevier (Netherlands)	0.95 (2015; calculated by Research	N/A	Yes	Quarterly	Corresponding author from an institution based in countries in Group A and Group B of the Hinari list may receive full waiver of

			Gate)				50% discount, respectively
Journal of Global Health Perspectives	N/A	First Aid WorldWide (Menlo Park, CA)	N/A	N/A	Yes	No schedule	Not reported
Global Health: Science and Practice (GHSP)	Supported by USAID's Office of Population and Reproductive Health, Bureau for Global Health	John Hopkins Center for Communication Programs (USA)	2.65 (2015; calculated by Research Gate)	N/A	Yes	Quarterly	No publication fees charged to authors
Integrative Journal of Global Health	N/A	Insight Medical Publishing/iMedPub LTD (UK)	N/A	N/A	Yes	3 times a year	No discount or waived fees policy

International Health

**Journal name**	**Affiliation**	**Publisher**	**Impact factor**	**5-year impact factor**	**Open access**	**Frequency of publication**	**Financial support for publication fees**
International Health	Royal Society of Tropical Medicine and Hygiene	Oxford Academic/Oxford University Press (UK)	1.784	1.757	Options to publish open access ($3150 general articles; reduced for developing countries)	6 times per year	Waivers apply for corresponding authors from LIC and MIC and those in genuine hardship
International Journal Health Services	N/A	SAGE Journals (UK)	1.135	1.215	Some articles have open access but there are also	Quarterly	No fees payable to publish

					subscription options		
Tropical Medicine & International Health	London School of Hygiene & Tropical Medicine, Swiss Tropical and Public Health Institute, Foundation Tropical Medicine and International Health Amsterdam, Netherlands,	John Wiley & Sons (founded in USA but distribution worldwide)	2.85	N/A	Yes option available	Monthly	No page charges for all authors

	Belgian Institute of Tropical Medicine and Bernhard-Nocht-Institute for Tropical Medicine						
International Journal of Healthcare	N/A	Sciedu Press (Canada)	N/A	N/A	Yes	Twice a year	No waived fees or discounts available
International Journal of Health Policy and Management	N/A	Kerman University of Medical Sciences (Iran)	1.36 (2015; from Research Gate)	N/A	Yes	Monthly	No submission or publication fees
International	N/A	Taylor & Francis	0.39 (2015;	N/A	Yes	Quarterly	No publication fees or

Journal of Healthcare Management		Online (England & Wales)	from Research Gate)				page charges for journal
International Journal of Medical Sciences	N/A	Ivyspring International Publisher (Australia)	0.517	N/A	Yes	Monthly	No policy on discount or waived publication fees
